# Silymarin attenuates oxidative, inflammatory and apoptotic damage induced by a fungicide mixture in rat liver, kidney and brain

**DOI:** 10.1016/j.toxrep.2026.102287

**Published:** 2026-06-05

**Authors:** Hamada S. Salem, Asmaa M.R. Gouda, Nesreen M. Essam, Hanaa T. El-Bahnasy

**Affiliations:** Department of Zoology, Faculty of Science, Mansoura University, 35516, Egypt

**Keywords:** Hepatoprotection, Nephroprotection, Neuroprotection, Inflammation, Silymarin, Fungicide toxicity, Oxidative stress

## Abstract

Fungicide exposure is associated with significant health hazards in both animals and humans. The present study examined the toxicological effects of a mixture of copper oxychloride, propiconazole, boscalid, and pyraclostrobin on the liver, kidney, and brain of male rats, and evaluated whether silymarin could counteract fungicide-associated injury. Forty male Sprague–Dawley rats were randomly allocated to four groups: control, fungicide-treated, silymarin-treated, and fungicide and silymarin-treated. A panel of biochemical indices, oxidative stress measures, and inflammatory and apoptotic markers was quantified. In fungicide-treated rats, serum liver enzymes, bilirubin, urea, creatinine, and uric acid were higher than in controls. Total protein, neurochemical, and electrolyte markers also decreased significantly. Fungicide exposure increased malondialdehyde and lowered total antioxidant capacity and other antioxidant defences; at the same time, inflammatory and apoptotic markers rose. When silymarin was given alongside the fungicide mixture, it substantially dampened these biochemical disturbances and strengthened antioxidant status; inflammatory and apoptotic responses were also reduced. In conclusion, the findings support the potential use of silymarin supplements as an additional protective agent against chemical toxicity.

## Highlights

• Silymarin protected liver, kidney and brain from 4-fungicide mixture in rats.
• Co-treatment normalized ALT/AST/ALP, bilirubin and improved renal markers.
• Silymarin restored TAC, SOD, CAT and GSH while lowering MDA, NO and PC.
• Inflammation/apoptosis (TNF-α, IL-6, NF-κB, caspase-3, cyto c) were reduced.
• Histology confirmed reduced fatty change, nephritis and neuronal ischemic injury.

## Graphical Abstract

**Figure:**
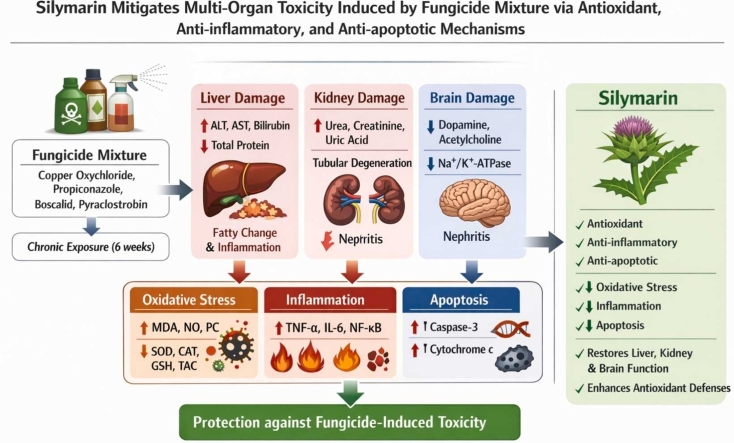

## 1 Introduction

Fungicides have become essential in modern agriculture and horticulture [1]. They suppress fungal pathogens or eliminate them, helping prevent crop infections. However, the growing reliance on these chemicals has raised major concerns about health and environmental impacts [1], and some can bioaccumulate through the food chain into fish and other animal species. Humans are exposed mainly by inhalation and ingestion as well as through dermal contact [2], [3]. Adverse biological effects on the body can result from various exposures to fungicides. The liver is the most affected organ by such adverse impacts [4], [5], [6], but secondary effects on the kidney and brain cannot be ruled out due to the decreased liver efficiency, oxidative stress, inflammation and interconnected metabolic pathways.

Copper oxychloride, propiconazole, boscalid and pyraclostrobin are common fungicides in Egyptian agriculture practices as well as worldwide. According to the Egyptian Agricultural Pesticides Committee [7], all four fungicides are officially registered for use in Egypt, with overlapping crops, diseases, and seasons. They are frequently used sequentially or in combination (e.g., Boscalid and pyraclostrobin) as part of integrated disease-management programs [7]. For example, they are listed as alternative options for managing a tomato disease called the early blight [7].

Copper oxychloride effectively controls fungal diseases in many crops, such as cucumbers, grapes, tomatoes and potatoes [8]. Recent research has reported its harmful effects on non-target organisms; however, these harmful effects are not well studied in mammals. Copper oxychloride includes copper that can accumulate in various tissues, disturb enzyme activities [9] and cause reactive oxygen species (ROS)-mediated oxidative and cellular damage [10]. Propiconazole, a triazole fungicide, is routinely applied to suppress foliar diseases; it is used on vegetables, fruits, grasses, and seeds [11]. Experimental evidence associates propiconazole with oxidative stress, diminished antioxidant capacity and changes in protein structures [11], [12].

Pyraclostrobin is a fungicide increasingly used to control fungal infections in cereals, vegetables, fruits, and ornamental plants [13]. Although it is relatively non-persistent in the environment, which suggests lower toxicity to humans and animals [14], its widespread application raises growing concerns about potential health risks [13]. Recent studies have reported toxic effects on non-target organisms, including zebrafish [15] and rats [16], and have shown that pyraclostrobin can increase ROS production [13]. Boscalid is a carboximide fungicide widely used to control diseases in fruit trees, grapes, rapeseed, vegetables, and field crops. Its heavy use has led to frequent detection in aquatic environments [17]. Although studies have well characterised its toxicity to aquatic organisms such as zebrafish [18], [19], few studies have assessed toxic effects in mammals [20].

Although human exposure generally occurs at lower levels, repeated, sequential, or residue-based agricultural co-exposure to these fungicides represents a relevant hazard scenario. The widespread use of these four fungicides has increased concerns about adverse health effects. Therefore, research must identify supplements that mitigate fungicide-induced toxicity. In this context, silymarin has shown considerable potential to protect against chemically induced injuries. It is a flavonoid complex derived from the seeds of Silybum marianum and consists primarily of flavonolignans, including silibinin (silybin) and silydianin. The phenolic structure of these flavonolignans confers strong antioxidant activity, with silybin being the primary biologically active component [21], [22]. In addition to its antioxidant activity, silymarin exhibits anti-inflammatory, antifibrotic, and immunomodulatory effects [23], [24]. Despite these well-documented protective properties, relatively few studies have investigated the potential of silymarin to counteract fungicide-induced toxicity.

Therefore, this research explores the combined harmful effects of the selected mixture on the liver, kidney and brain. Serum biochemical, oxidative stress, inflammatory and apoptotic markers were measured to determine the net impact of these fungicides. The study also investigates the potential protective effects of silymarin against the harmful impact of these fungicides.

## 2 Materials and methods

### 2.1 Animal housing and maintenance

The experimental protocol was approved by the Mansoura University Animal Care and Use Committee, Egypt (Approval No. SC.R.24.11.20). This study was performed following the guidelines provided by the committee. Male rats (Sprague Dawley, 8 weeks and 150–165.7 g) were obtained from EGYVAC, Egypt and housed under controlled conditions of temperature, humidity and lighting in standard cages for two weeks before conducting the study. All experimental procedures were conducted in accordance with institutional ethical guidelines and followed the National Institutes of Health (NIH) guidelines for the care and use of laboratory animals [25].

### 2.2 Chemicals and reagents

Copper oxychloride (Copper Arikh 50% WP from International Company for Chemical Industries, Egypt), propiconazole (Zol Young 25% EC from Shanxi Hengtian Chemical Co. Ltd., China) and boscalid and pyraclostrobin (Bellis 38% WG from BASF Agricultural Solutions, Egypt) were obtained from a local merchant in Mansoura city. Silymarin 140 mg capsules (from Chemical Industries Development, Egypt) were acquired from a local pharmacy in Mansoura city. The rest of the chemicals were utilised with analytical grade. All the parameters were determined based on the manufacturer's instructions presented with the kits.

### 2.3 Experimental design

The experimental duration for this study was 6 weeks to mimic a repeated sub-chronic exposure model. Male rats were randomly divided into four groups, with ten rats in each group. The animals were treated throughout the study period as follows:

1. Control (C) Group: The control rats received standard laboratory food (SLF) and distilled water; they also received a vehicle control (corn oil; 5 mL/kg) three times per week.
2. Silymarin-treated (S) group: Animals in this group remained on SLF and distilled water while receiving silymarin at 100 mg/kg BW; it was administered five times per week.
3. Fungicide-treated (F) group: Rats in this group were given distilled water and SLF. Three times per week, they were administered corn oil (5 mL/kg) containing a mixture of four fungicides. This mixture included copper oxychloride (73.5 mg/kg BW; 1/20 LD50) [26], propiconazole (35 mg/kg BW; 1/40 LD50) [11], boscalid (60 mg/kg BW; 1/80 LD50) [27], and pyraclostrobin (30 mg/kg BW; 1/150 LD50) [16].
4. Fungicides and Silymarin-treated (FS) group: In this group, rats received distilled water, SLF, corn oil (5 mL/kg) that contains copper oxychloride (73.5 mg/kg BW), propiconazole (35 mg/kg BW), boscalid (60 mg/kg BW) and pyraclostrobin (30 mg/kg BW) 3 times per week. This group also received silymarin at 100 mg/kg BW; it was administered five times per week as scheduled.

All vehicle, fungicide, and silymarin administrations were performed by oral gavage using a stomach tube. The dose ratios were determined based on previous research [11], [16], [26], [27] and chosen as fixed fractions of the reported oral LD50 values for each compound to yield sublethal levels that allow sustained exposure while avoiding overt toxicity and mortality. This approach was used to mimic a low-dose, mixed-exposure scenario that could be relevant for agricultural workers and consumers chronically exposed to multiple residues, even though real-life human exposures generally occur at lower absolute doses.

### 2.4 Sampling and tissue collection

After the experiment, all rats underwent an overnight fast. They were then anaesthetised with ketamine and pentobarbital sodium before being euthanised. Blood was obtained, after which serum was separated and then stored at −80 °C for further testing. The liver, kidney and brain were quickly removed; then, after washing them with ice-cold saline solution, they were stored at −80 °C for further testing.

### 2.5 Biochemical analysis

The collected serum samples were used for the analysis of different biochemical markers. Commercial kits from Spin React, Spain were utilised to assess the concentration of alanine aminotransferase (ALT; catalogue number BEIS11-P), aspartate aminotransferase (AST; catalogue number MD41264), alkaline phosphatase (ALP; catalogue number MD41233) and total bilirubin (catalogue number MD1001042). Total protein content (Bio-Diagnostic, Egypt, catalogue number TP2020) was evaluated utilising the Biuret technique, according to Gornall et al. [28]. Serum renal function markers were estimated by measuring urea (Bio-Diagnostic, Egypt, catalogue number UR2110), uric acid (Bio-Diagnostic, Egypt, catalogue number UA2120), and creatinine (Bio-Diagnostic, Egypt, catalogue number CR1250) levels using respective commercial kits.

After homogenisation of different tissue samples in ice-cold phosphate buffer, they were centrifuged, and the supernatant was obtained. The levels of dopamine (Eagle Bioscience, USA, catalogue number DOU39-K01), acetylcholine (MyBioSource, USA, catalogue number MBS169077), sodium (MyBioSource, USA, catalogue number MBS2540574) and Na-K ATPase (MyBioSource, USA, catalogue number MBS8243226) were detected in the brain homogenates according to the manufacturer’s instructions.

The concentrations of the following oxidative stress parameters were assessed in homogenates utilising colourimetric kits acquired from Bio-Diagnostic, Egypt: malondialdehyde (MDA; catalogue number MD 25 29), superoxide dismutase (SOD; catalogue number SD 25 21), catalase (CAT; catalogue number CA 25 17) and glutathione (GSH; catalogue number GR 25 11). Total antioxidant capacity (TAC; Bio-Diagnostic, Egypt, catalogue number TA 25 13) was evaluated spectrophotometrically following the approach of Benzie and Strain [29]. Levels of nitric oxide (NO; Bio-Diagnostic, Egypt, catalogue number NO 25 33) and protein carbonyl (PC; MyBioSource, USA, catalogue number MBS2600784) were measured in the brain homogenates according to the manufacturer’s instructions.

Furthermore, inflammatory markers were measured in the tissue homogenates. Utilising enzyme-linked immunosorbent assay (ELISA) kits, levels of tumour necrosis factor-alpha (TNF-α; Cusabio, USA, catalogue number CSB-E11987r), nuclear factor-kappa B (NF-κB; Cusabio, USA, catalogue number CSB-E12107h) and interleukin−6 (IL−6; Cusabio, USA, catalogue number CSB-E04640r) were quantified following the instructions of the manufacturer. Markers of apoptosis were determined in the homogenates. Caspase−3 levels were assessed using a kit from Biovision, USA (catalogue number E4592–100). Cytochrome c release from mitochondria was detected using an ELISA kit from Cusabio, USA (catalogue number CSB-EL006328RA) according to the supplier’s protocol.

### 2.6 Histopathological analysis

Specimens of the liver, kidney and brain were isolated and immediately kept in 10% neutral formaldehyde. Next, these samples were embedded in paraffin. They were then sectioned at a thickness of 5 μm by using a microtome and then mounted on dry and clean glass slides. Finally, all tissue samples were stained with Hematoxylin and Eosin. The stained sections were checked by an Olympus light microscope.

### 2.7 Statistical analysis

All biological samples for each parameter (n = 4) were determined in technical triplicate. All data were presented as Mean ± SD. The data obtained from the biochemical analysis, including function markers, oxidative stress markers, and inflammation and apoptosis markers, were analysed utilising SPSS Software (version 20). Differences in the data generated were statistically tested using one-way analysis of variance (ANOVA) followed by post-hoc Tukey test. In the study, a result was deemed statistically significant if the *p*-value was less than 0.05.

## 3 Results

This research assesses the potential protective effects of silymarin versus the toxicity caused by a mixture of fungicides (copper oxychloride, propiconazole, boscalid and pyraclostrobin) in male rats.

### 3.1 Biochemical function markers

Table 1 reports liver biochemical markers for the four groups. No statistically significant differences emerged between the C and S groups. In the F group, exposure to the fungicide mixture significantly increased AST, ALT, and ALP relative to the C group (p < 0.05). When silymarin was given with the fungicides, AST, ALT, and ALP in the FS group were significantly lower than in the F group (p < 0.05). Bilirubin also changed, rising in the F group compared with the C group (p < 0.05); this rise was significantly countered in the FS group (p < 0.05). Total protein decreased significantly in the F group (p < 0.05). In the FS group, co-treatment with silymarin maintained total protein at levels comparable to the C group, and it was significantly higher than in the F group (p < 0.05).

**Table 1:** Serum AST, ALP, ALT, total protein levels and bilirubin concentrations in different groups.

**Parameters**	**Animal groups**			
	**C**	**S**	**F**	**FS**
ALT (U/L)	37.60 ± 3.72	35.00 ± 6.32	77.20^a^ ± 12.18	50.40^b^ ± 10.42
AST (U/L)	33.00 ± 5.56	33.20 ± 4.70	164.00^a^ ± 20.22	81.80^ab^ ± 20.36
ALP (U/L)	135.00 ± 10.98	133.70 ± 4.28	177.00^a^ ± 4.18	153.20^ab^ ± 11.38
Bilirubin (mg/dl)	0.90 ± 0.10	0.88 ± 0.18	1.36^a^ ± 0.38	0.87^b^ ± 0.04
Total protein (g/dl)	6.82 ± 0.52	6.83 ± 0.42	5.31^a^ ± 0.74	6.83^b^ ± 0.44

*Note.* Results are means ± SD (standard deviation; n = 4 biological replicates per group). C: Control; S: Silymarin; F: Fungicides; FS: Fungicides and Silymarin. (a): Significant compared with the C group (p < 0.05). (b): Significant compared with the F group (p < 0.05).

Table 2 presents renal function markers across the groups. Between the C and S groups, no significant differences were detected for urea, creatinine and uric acid. In the F group, all three markers significantly increased relative to the C group (p < 0.05). Silymarin co-administration reduced creatinine, urea, and uric acid in the FS group compared with the F group (p < 0.05).

**Table 2:** Serum urea, creatinine, and uric acid levels in different groups.

**Parameters**	**Animal groups**			
	**C**	**S**	**F**	**FS**
Urea (mg/dl)	34.00 ± 7.75	33.75 ± 5.44	67.00^a^ ± 6.06	44.75^b^ ± 5.12
Creatinine (mg/dl)	0.90 ± 0.22	0.96 ± 0.25	1.92^a^ ± 0.32	1.11^b^ ± 0.32
Uric acid (mg/dl)	3.77 ± 0.10	4.48 ± 0.93	7.71^a^ ± 1.64	4.99^b^ ± 1.34

*Note.* Results are means ± SD (standard deviation; n = 4 biological replicates per group). C: Control; S: Silymarin; F: Fungicides; FS: Fungicides and Silymarin. (a): Significant compared with the C group (p < 0.05). (b): Significant compared with the F group (p < 0.05).

Table 3 summarises neurochemical and electrolyte outcomes across the four groups. There were no statistically significant differences between the C and S groups. Rats in the F group exhibited significant decreases (*p* < 0.05) in dopamine, acetylcholine, sodium (Na+), and Na-K ATPase activity versus the C group. With silymarin given alongside the fungicides, those decreases were less pronounced; the FS group showed significant alleviation compared with the F group (p < 0.05).

**Table 3:** Brain dopamine, acetylcholine, sodium levels and Na-K ATPase activity in different groups.

**Parameters**	**Animal groups**			
	**C**	**S**	**F**	**FS**
Dopamine (ng/mg)	1.53 ± 0.16	1.61 ± 0.14	0.72^a^ ± 0.29	1.19^b^ ± 0.07
Acetylcholine (ng/mg)	4.66 ± 0.50	4.60 ± 0.62	1.14^a^ ± 0.01	2.35^ab^ ± 0.14
Brain Na + (mmol/L)	150.30 ± 4.67	159.00 ± 5.03	129.00^a^ ± 5.03	139.00^ab^ ± 2.31
Na-K ATPase (ng/mg)	1.76 ± 0.18	1.87 ± 0.34	0.91^a^ ± 0.10	1.49^b^ ± 0.17

*Note.* Results are means ± SD (standard deviation; n = 4 biological replicates per group). C: Control; S: Silymarin; F: Fungicides; FS: Fungicides and Silymarin. (a): Significant compared with the C group (p < 0.05). (b): Significant compared with the F group (p < 0.05).

### 3.2 Antioxidants and oxidative stress parameters

Table 4 reports oxidative stress markers in the liver, kidney, and brain for all four groups. The C and S groups did not differ significantly; their values were relatively similar. Relative to the C group, the F group showed significantly higher MDA and lower TAC, SOD, CAT, and GSH in the liver, kidney, and brain (p < 0.05). In the brain, the F group also had significantly higher PC and NO levels (p < 0.05). In the FS group, silymarin reduced MDA and increased TAC, SOD, CAT, and GSH in the liver, kidney, and brain relative to the F group (p < 0.05). Brain PC and NO were significantly lower in the FS group than in the F group (p < 0.05).

**Table 4:** CAT, SOD, MDA, GSH and TAC concentrations in the liver, kidney and brain and NO and PC in the brain in the four different groups.

**Parameters**		**Animal groups**			
		**C**	**S**	**F**	**FS**
TAC (mM/g)	Liver	277.00 ± 11.62	278.50 ± 14.66	217.60^a^ ± 25.48	257.80^b^ ± 7.26
	Kidney	275.10 ± 16.16	276.10 ± 19.15	210.70^a^ ± 17.59	244.60^b^ ± 5.50
	Brain	222.00 ± 11.20	223.50 ± 14.80	173.00^a^ ± 20.80	206.00^b^ ± 6.00
MDA (nmol/g)	Liver	732.30 ± 35.66	730.80 ± 21.89	982.60^a^ ± 22.54	913.70^ab^ ± 36.20
	Kidney	758.00 ± 43.12	757.10 ± 28.04	975.90^a^ ± 27.76	889.00^ab^ ± 58.50
	Brain	743.10 ± 26.96	736.80 ± 15.17	951.80^a^ ± 25.58	896.20^ab^ ± 13.30
SOD (U/g)	Liver	173.30 ± 8.14	172.90 ± 12.00	124.60^a^ ± 9.10	146.80^ab^ ± 8.38
	Kidney	171.30 ± 13.14	174.00 ± 8.63	132.40^a^ ± 5.43	154.60^b^ ± 10.72
	Brain	186.80 ± 7.53	188.30 ± 13.01	128.30^a^ ± 4.39	152.40^ab^ ± 4.47
CAT (U/g)	Liver	175.50 ± 14.55	176.30 ± 10.33	123.90^a^ ± 9.57	146.70^ab^ ± 7.44
	Kidney	183.40 ± 5.78	177.20 ± 11.06	132.20^a^ ± 5.89	152.00^ab^ ± 9.51
	Brain	147.00 ± 8.94	148.00 ± 8.78	107.00^a^ ± 9.61	132.0^b^ ± 10.39
GSH (µmol/g)	Liver	5.03 ± 0.58	5.08 ± 0.56	2.30^a^ ± 0.32	3.39^ab^ ± 0.55
	Kidney	5.01 ± 0.60	4.77 ± 0.80	1.91^a^ ± 0.11	3.08^ab^ ± 0.46
	Brain	4.75 ± 0.43	4.79 ± 0.16	2.03^a^ ± 0.31	2.94^ab^ ± 0.22
NO (µmol/g)	Brain	19.70 ± 0.69	18.71 ± 0.60	47.80^a^ ± 6.71	36.38^ab^ ± 5.30
PC (nmol/g)	Brain	0.91 ± 0.08	0.87 ± 0.05	4.97^a^ ± 0.85	2.67^ab^ ± 0.47

*Note.* Results are means ± SD (standard deviation; n = 4 biological replicates per group). C: Control; S: Silymarin; F: Fungicides; FS: Fungicides and Silymarin. (a): Significant compared with the C group (p < 0.05). (b): Significant compared with the F group (p < 0.05).

### 3.3 Inflammatory and apoptotic markers

Table 5 illustrates inflammatory and apoptotic markers in the liver, kidney and brain of the four studied groups. No significant differences were observed between the C and S groups. The findings revealed that the F group exhibited significantly (*p* < 0.05) higher concentrations of TNF-α, IL−6, caspase 3, NF-κB and cytochrome c compared with the C group. The FS group showed significant (*p* < 0.05) reductions in TNF-α, IL−6, caspase 3, NF-κB and cytochrome c levels compared with the F group.

**Table 5:** IL−6, TNF-α, NF-κB, Caspase 3, and cytochrome c concentrations in different groups.

**Parameters**		**Animal groups**			
		**C**	**S**	**F**	**FS**
TNF-α (pg/mg)	Liver	14.42 ± 1.12	13.86 ± 0.81	32.79^a^ ± 4.98	23.54^ab^ ± 4.61
	Kidney	21.53 ± 3.64	21.70 ± 2.08	46.50^a^ ± 7.46	30.43^b^ ± 8.67
	Brain	22.15 ± 1.75	21.43 ± 2.28	49.14^a^ ± 6.50	36.50^ab^ ± 4.98
IL−6 (pg/mg)	Liver	4.75 ± 0.84	5.00 ± 0.95	11.64^a^ ± 2.17	7.82^ab^ ± 0.52
	Kidney	4.51 ± 0.63	4.32 ± 0.81	9.94^a^ ± 1.95	6.57^b^ ± 0.54
	Brain	4.94 ± 1.05	4.89 ± 0.97	11.34^a^ ± 1.84	8.02^ab^ ± 0.39
**NF-κB (pg/mg)**	Liver	20.30 ± 1.50	20.05 ± 1.56	34.90^a^ ± 3.73	29.20^ab^ ± 3.23
	Kidney	19.68 ± 1.37	19.79 ± 1.61	32.10^a^ ± 5.11	23.79^b^ ± 1.55
	Brain	20.85 ± 2.55	20.12 ± 1.23	40.40^a^ ± 2.64	32.00^ab^ ± 5.41
Caspase 3 (ng/mg)	Liver	0.83 ± 0.10	0.85 ± 0.08	7.14^a^ ± 1.70	2.38^b^ ± 0.54
	Kidney	0.83 ± 0.10	0.77 ± 0.16	7.91^a^ ± 0.67	4.39^ab^ ± 0.72
	Brain	1.17 ± 0.31	1.25 ± 0.46	9.36^a^ ± 1.87	7.07^ab^ ± 0.53
Cytochrome C (pg/mg)	Liver	155.80 ± 8.07	152.70 ± 11.00	274.50^a^ ± 15.66	248.20^ab^ ± 12.25
	Kidney	180.00 ± 12.73	181.80 ± 2.83	257.10^a^ ± 23.40	202.80^b^ ± 11.50
	Brain	186.60 ± 8.57	183.50 ± 7.85	258.90^a^ ± 12.53	232.10^ab^ ± 16.17

*Note.* Results are means ± SD (standard deviation; n = 4 biological replicates per group). C: Control; S: Silymarin; F: Fungicides; FS: Fungicides and Silymarin. (a): Significant compared with the C group (p < 0.05). (b): Significant compared with the F group (p < 0.05).

### 3.4 Histopathological results

**Fig. 1:**
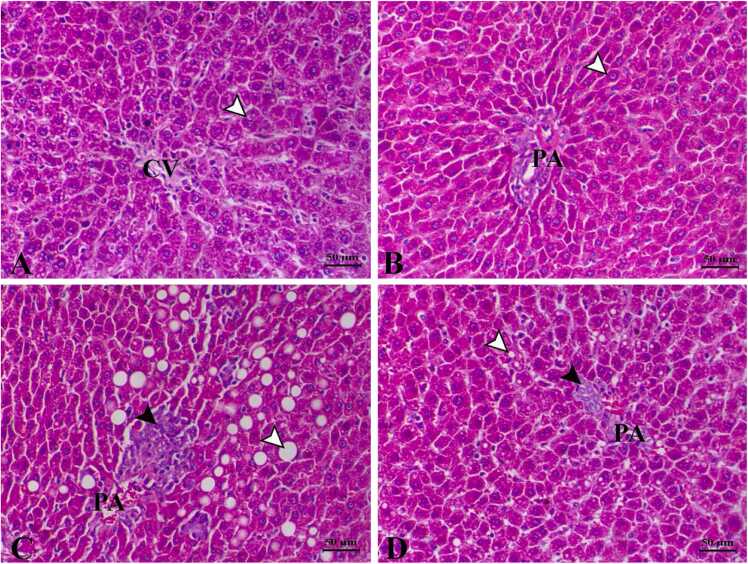
Liver histology. (A) Control: normal hepatocytes arranged in cords around the central vein (CV). (B) Silymarin: normal hepatocytes around the portal area (PA). (C) Fungicide: marked hepatocellular vacuolation/fatty change with marked periportal mononuclear inflammation (PA). (D) Fungicides and silymarin: mild fatty change with mild bile duct epithelial hyperplasia (PA). H&E, × 200; scale bar = 50 µm.

**Fig. 2:**
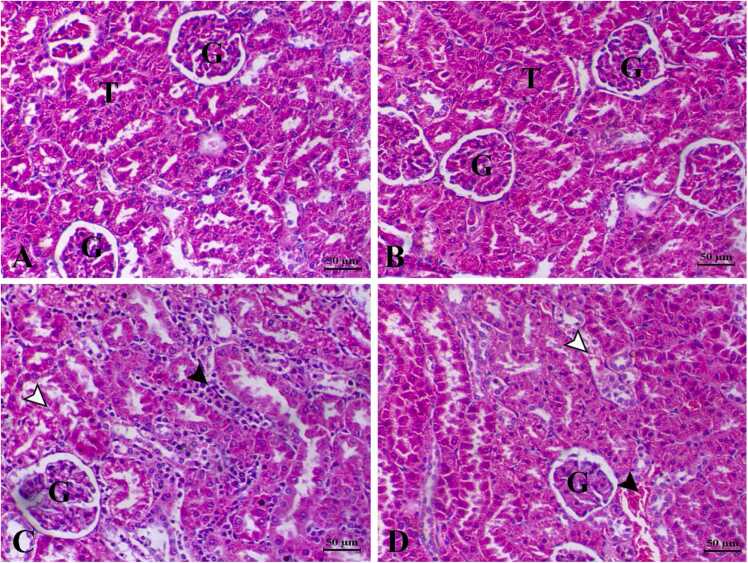
Kidney histology. (A) Control: normal renal parenchyma with normal glomeruli (G) and tubules (T). (B) Silymarin: normal glomeruli (G) and tubules (T). (C) Fungicide: interstitial nephritis with marked tubular degeneration and mononuclear inflammatory infiltration (lymphocytes/macrophages). (D) Fungicides and silymarin: markedly reduced interstitial inflammation with focal tubular degeneration and mild capillary congestion. H&E; scale bar = 50 µm.

**Fig. 3:**
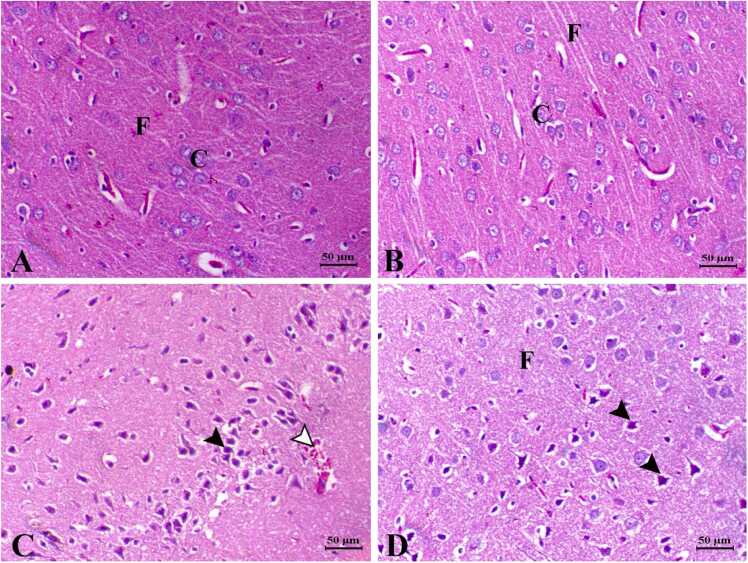
Brain (cerebral cortex) histology. (A) Control: normal neurons (C) and nerve fibres (F). (B) Silymarin: normal neurons (C) and fibres (F). (C) Fungicide: congested capillaries with severe ischemic neuronal changes (deep cytoplasmic basophilia and marked atrophy). (D) Fungicides and silymarin: markedly reduced ischemic neuronal injury. H&E, × 200; scale bar = 50 µm.rates per group). C: Control; S: Silymarin; F: Fungicides; FS: Fungicides and Silymarin. (a): Significant compared with the C group (p < 0.05). (b): Significant compared with the F group (p < 0.05).

Liver sections from the C and S groups showed normal hepatocyte cords around the central vein (Fig. 1). In the F group, the liver showed marked vacuolation with fatty change and periportal inflammation; mononuclear inflammatory cells infiltrated the tissue. In the FS group, the liver showed only mild fatty changes and mild hyperplasia of the bile duct epithelium (Fig. 1). Renal sections from the C and S groups showed normal parenchyma with intact glomeruli and tubules (Fig. 2). In the F group, renal tissue showed interstitial nephritis with pronounced tubular degeneration; mononuclear inflammatory cells, including lymphocytes and macrophages, infiltrated the interstitium. In the FS group, inflammation decreased substantially; only focal tubular degeneration and mild capillary congestion were observed (Fig. 2). The cerebral cortex of the C and S groups showed normal neuronal architecture with intact nerve fibres (Fig. 3). In the F group, capillary congestion was evident, and neurons showed severe ischemic changes; deep cytoplasmic basophilia and marked neuronal atrophy were present. In the FS group, these ischemic changes were substantially attenuated; the findings indicate potential protective effects of silymarin (Fig. 3).

## 4 Discussion

Fungicides play an important role in protecting crops from fungal infections [1]. However, the persistent application of fungicides leaves harmful residues in water and food, which may cause severe health problems, including liver disorders, for animals and humans [11]. After fungicide residues enter the body, their main target with the greatest risk of harmful impact is the liver [30]. Secondary effects on the kidney and brain can occur due to decreased liver efficiency, oxidative stress, inflammation and interconnected metabolic pathways.

This study focused on four common fungicides that are frequently used in agriculture practices in Egypt. Although studies have investigated their harmful effects on aquatic organisms, limited research exists on their impact on mammals. Moreover, the combined toxic effect of being exposed to sub-lethal levels of these fungicides on non-target animals remains understudied. In real-world environments, animals and humans are often subjected to multiple toxins at the same time. Even at very small doses, when combined, they may result in significant harm, especially for consumers and farm workers. Thus, research is required to identify effective supplements that could provide protection for humans against exposure to such chemicals. Silymarin has shown potential effects in protecting the liver from various environmental toxic chemicals [23], [31]. Therefore, this research evaluates the potential protective effects of silymarin against toxicity caused by copper oxychloride, propiconazole, boscalid and pyraclostrobin in rats.

In this study, the fungicides were tested for the induction of hepatotoxicity by the estimation of hepatic biochemical markers that are released into the blood. The present study displayed a significant rise in the concentrations of AST, ALP, ALT and bilirubin and a significant decline in the concentration of total protein in the fungicide-treated group compared with rats of the control group. The concentrations of these parameters significantly change in cases of acute or mild liver injuries [31]. These changes may be attributed to negative changes in the permeability of the tissues, cell fragmentation, or cellular injuries [31] caused by the hepatotoxic impact of the studied fungicide mixture. Previous reports obtained similar results in rodents and aquatic animals after exposure to copper oxychloride [26], [31], [32], pyraclostrobin [16], [33], propiconazole [11] and boscalid [34].

Renal function markers were assessed across the studied groups. Elevated urea, creatinine, and uric acid in fungicide-exposed rats indicate substantial cellular breakdown [32]. Such elevations suggest fungicide-related renal injury that undermines filtration and tubular functions; therefore, the kidneys excrete these products less effectively. Earlier studies on copper oxychloride and propiconazole reported impaired renal function under these exposures [11], [26], [32], [35].

Significant decreases were observed in acetylcholine, dopamine, Na⁺, and Na⁺/K⁺-ATPase activity; together, these findings indicate broad neurochemical and electrolyte disruption. Reduced acetylcholine can impair learning and memory [36]. Na⁺/K⁺-ATPase is a membrane-bound enzyme that maintains transmembrane ionic gradients by exporting Na⁺ and importing K⁺ after depolarisation [37]. Fungicide-associated oxidative stress may have contributed to Na⁺/K⁺-ATPase inhibition, because the enzyme is highly sensitive to free radical damage [37]. When this pump slows, cells lose ionic balance; this change can promote neuronal death and impair learning and memory processes. These neurochemical changes may reflect oxidative stress, mitochondrial dysfunction, and neuroinflammation after fungicide exposure; this interpretation aligns with reported mechanisms in related models [26], [38]. Prior work reports neurobehavioral toxicity with propiconazole [38], brain tissue degeneration with copper oxychloride [26], pyraclostrobin-driven mitochondrial dysfunction in zebrafish neurons leading to energy depletion and neuronal death [39], and boscalid-associated neurodevelopmental defects in zebrafish [40].

However, silymarin partially mitigated disturbances in liver, kidney, and brain functions caused by fungicides. These results confirm the protective potential of silymarin against chemical-induced injuries. Numerous studies support that silymarin possesses protective effects against toxic agents in tissues, including the liver, kidney and brain. For example, Wu et al. [41] described that silymarin is able to mitigate liver and brain damage in rats exposed to difenoconazole, a triazole fungicide. Similarly, one study found that silymarin protects the mouse liver and kidney from thioacetamide-induced toxicity [42].

To explore the biological changes associated with fungicide-induced tissue damage, markers of oxidative stress, inflammation, and apoptosis were analysed. In this study, exposure to the fungicide mixture significantly reduced TAC, SOD, CAT, and GSH levels while markedly increasing MDA concentrations in the liver, kidney, and brain. Elevated NO and protein carbonyl (PC) levels were also detected in the brain. These findings indicate oxidative imbalance and molecular damage in the examined tissues. Similar oxidative disruptions have been reported following exposure to pyraclostrobin [16], propiconazole [11], [33], [38], [43], [44], copper oxychloride [8], [26], [31], [35], and boscalid [40], [45], [46]. Therefore, oxidative stress is considered a plausible contributor to the observed toxicity.

Conversely, silymarin treatment was associated with a significant reduction in oxidative stress markers, indicated by lower NO and PC in the brain, increased TAC, SOD, CAT and GSH, and reduced MDA concentrations in the three studied tissues. These findings support the antioxidant potential of silymarin and suggest that it may attenuate fungicide-associated oxidative imbalance. Researchers found that silymarin safeguards tissues from oxidative damage induced by maneb and paraquat [47], diclofenac [48], paracetamol [23] and difenoconazole [41]. The antioxidant mechanism of silymarin has been proposed to involve free-radical scavenging and inhibition of lipid peroxidation (LPO) [23], [41]. It may also help preserve cell membranes and support ribosomal and RNA synthesis [41].

A close relationship between oxidative stress and inflammation is recognised, as these processes can influence each other [49]. Therefore, IL−6, NF-κB and TNF-α, key inflammatory markers, were analysed in the studied rats. TNF-α is known to interact with NF-κB-dependent inflammatory signalling, which can promote the expression of pro-inflammatory cytokines, including IL−6. Elevated IL−6 levels can contribute to persistent inflammation. In this study, increased TNF-α, IL−6 and NF-κB protein levels were observed in the liver, kidney and brain of fungicide-treated rats. High inflammatory marker levels were also reported in previous studies after exposure to pyraclostrobin [16], boscalid [18], [20], copper oxychloride [31], [32] and propiconazole [50]. Inflammation is also closely linked to apoptosis, which can occur when the balance between anti-apoptotic and pro-apoptotic signals shifts toward cell death [51]. In the present study, fungicide-exposed rats showed increased caspase−3 and cytochrome c levels. These findings are consistent with apoptotic-related changes. Similar apoptotic marker responses have been documented previously in studies conducted with boscalid [18], copper oxychloride [31], [32], propiconazole [11] and pyraclostrobin [13], [16].

Administration of silymarin had a partial protective effect against these alterations. The concentrations of TNF-α, IL−6, NF-κB, caspase 3 and cytochrome c were decreased as compared with the group treated with only fungicides, but the response was not uniformly restored to control values. These results suggest that silymarin partially attenuated inflammatory and apoptotic marker changes associated with fungicide-induced tissue damage. The suppression of inflammasome activation is one proposed mechanism through which silymarin may exert anti-inflammatory activity, as reported by Abed et al. [23] and Guo et al. [52]. Also, silymarin is shown to decrease the production of TNF-α [23]. Silymarin treatment has also been demonstrated to significantly inhibit the pro-inflammatory cytokine IL−6 [52]. It was also shown that silymarin could prevent TNF-α-induced apoptosis and inhibit the expression of apoptotic proteins, such as Bax and caspase 3 [53], [54].

The histopathological findings corroborated the biochemical results, showing significant tissue alterations in fungicide-treated rats. In the liver, fungicide exposure produced hepatic vacuolation, fatty changes, and periportal inflammation, consistent with previous reports on copper oxychloride [55], propiconazole [56], boscalid [57], and pyraclostrobin [16]. The brain also exhibited severe ischemic changes, including neuronal atrophy and capillary congestion, in agreement with earlier studies on propiconazole [58] and copper oxychloride [8], [26]. Renal sections from fungicide-treated rats showed interstitial nephritis, tubular degeneration, and inflammatory cell infiltration, findings that align with previous observations for copper oxychloride [32], propiconazole [11], and pyraclostrobin [59]. These pathological alterations were substantially reduced in the fungicides and silymarin group, consistent with results reported by Heidarian and Nouri [48]. Collectively, these findings support the conclusion that silymarin partially preserves normal tissue architecture and mitigates the adverse effects of fungicide exposure.

Given the widespread use of these fungicides, the implications of these findings are particularly relevant for public health. The findings support the need to consider pesticide mixtures in safety assessments, as humans may experience repeated or sequential exposure to multiple fungicides through crop-management practices, residues, and occupational contact. The results may therefore inform future regulatory and monitoring strategies that address combined exposures rather than single-compound toxicity alone [7], [25].

However, several limitations should be considered. First, because individual fungicide groups were not included, the study cannot determine whether the observed toxicity was additive, synergistic, antagonistic, or driven mainly by one compound. In addition, although LD_50_-based dosing is useful for controlled toxicological hazard identification, it does not reflect the lower and more variable exposures expected from dietary residues, environmental contamination, or occupational contact. Future studies should include residue-relevant doses, comparisons across exposure routes, and longer experimental durations to better model human and environmental exposure scenarios. Additionally, further research should clarify the pathways underlying fungicide toxicity and the protective effects of silymarin. A limitation of this study is that although Tukey’s post-hoc test controlled pairwise comparisons within each endpoint, no global correction was applied across all markers, which may increase the risk of type I error. Future studies should include predefined primary endpoints and appropriate multiple-testing correction methods. Finally, future studies with larger sample sizes are required to support the findings of this research.

## 5 Conclusion

In conclusion, the results of this study highlight the adverse biological effects associated with repeated sub-chronic exposure to the tested fungicide mixture. Combined exposure was associated with biochemical dysfunction, oxidative imbalance, inflammatory-marker elevation and apoptotic-marker changes in the liver, kidney and brain. The results also support the potential mitigating effect of concurrently administered silymarin against fungicide-associated alterations in the liver, kidney and brain. Finally, the study supports the need for further research into safe, natural supplements such as silymarin that may offer partial protection against hepatic, renal and brain-related toxic effects of environmental chemical mixtures.

## CRediT authorship contribution statement

**Hamada S. Salem:** Writing – original draft, Visualization, Validation, Supervision, Methodology, Investigation, Data curation, Conceptualization. **Gouda Asmaa M. R.:** Writing – review & editing, Methodology. **Hanaa T. El-Bahnasy:** Writing – review & editing, Visualization, Validation, Software, Methodology, Investigation, Formal analysis, Data curation, Conceptualization. **Nesreen M. Essam:** Writing – review & editing, Methodology.

## Ethical statement

All experimental procedures were approved by the Mansoura University Animal Care and Use Committee (MU-ACUC), Egypt (Approval No. SC.R.24.11.20).

## Declaration of Competing Interest

The authors declare that they have no known competing financial interests or personal relationships that could have appeared to influence the work reported in this paper.

## Data Availability

Data will be made available on request.
